# Benign proliferative breast diseases among female patients at a sub Saharan Africa tertiary hospital: a cross sectional study

**DOI:** 10.1186/1471-2482-13-9

**Published:** 2013-04-01

**Authors:** Christopher Okoth, Moses Galukande, Josephat Jombwe, Dan Wamala

**Affiliations:** 1 Department of Surgery, School of Medicine, College of Health Sciences, Makerere University, P. O. Box 7072, Kampala, Uganda; 2 Department of Surgery, Mulago National Referral Hospital, Kampala, Uganda; 3 Department of Pathology, Makerere University College of Health Sciences, Kampala, Uganda

**Keywords:** Benign proliferative breast, Atypia, Breast cancer

## Abstract

**Background:**

Non-cancerous diseases of the breast have assumed increasing importance because of the public awareness of breast cancer. These benign diseases are a recognized important risk factor for later breast cancer which can develop in either breast. The risk estimate of these benign breast diseases has not been well established in sub Saharan Africa. Women with benign proliferative or atypical breast lesions have a two- fold risk of developing breast cancer in western populations. The purpose of this study therefore was to determine the prevalence of proliferative disease ( BPBD) with and without atypia among Ugandan Black women.

**Methods:**

A cross-sectional descriptive study conducted at Mulago Hospital Breast Clinic between January 2012 and June 2012; 208 women aged 12 years and above with palpable breast lumps were screened. Fine needle aspiration biopsies were taken for cytological examination.

**Results:**

Of the 208 women with benign breast lumps screened, 195 were recruited in the study. The prevalence of BPBD was 18% (35/195). BPBD with atypia was 5.6% (11/195). The mean age and body mass index (BMI) were 28.4 years and 23.26 kg/m^2^ respectively. The commonest lesions were fibroadenomas for 57%, (111/195), and fibrocystic change were 21% (40/195). Most BPBD with atypia lesions were in the fibrocystic category.

**Conclusions:**

Benign proliferative breast diseases are common, found mostly among premenopausal women. A significant proportion of BPBD had atypical proliferation. An accurate breast cancer risk estimate study for BPBD is recommended.

## Background

Non-cancerous diseases of the breast have assumed increasing importance in recent times because of the public awareness of breast cancer. The vast majority of the lesions that occur in the breast are benign [1-3].

Globally, benign pathological states account for approximately 90% of the clinical presentations related to the breast [4]. In states such as Uganda, Trinidad and Nigeria, benign breast diseases constituted 70-79% of breast lumps and these were mostly fibroadenoma and fibrocystic change [5-8]. Fibroadenoma, fibrocystic change and breast abscesses account for most of these benign lesions [8]. Benign breast disease is an important risk factor for a later breast cancer, which can develop in either breast [9].

It is therefore important for surgeons, pathologists and oncologists to recognize benign lesions, both to distinguish them from in situ and invasive breast cancer and to assess a patient’s risk of developing breast cancer, so that the most appropriate treatment modality for each case can be established [1].

Benign breast diseases encompasses a spectrum of histologic entities usually sub divided into non-proliferative breast lesions, proliferative breast lesions without atypia, and proliferative breast lesions with atypia [9,10]. The aim of this study was to determine the proportion of benign breast lesions that were proliferative and with or without atypia among black Ugandan women.

## Methods

### Study design

Cross-sectional descriptive study.

### Study setting

This study was conducted at the National Referral Hospital Breast Clinic, at Mulago in Kampala, Uganda which is conducted every Wednesday of the week.

### Study period

January to June 2012 inclusive.

### Participants

All female patients who were 12 years and above with palpable benign breast lump(s) and presented during the study period were eligible.

Those with breast lumps in pregnancy, ulcerated skin overlying the lumps and malignant lumps were excluded.

### Sample size estimation

The calculated sample size of 193 was taken to be the minimum required to answer all objectives.

### Study procedure and data collection

Fine needle aspiration biopsy cytology (FNAC) a minimally invasive technique of diagnosis pioneered by Torsten Lowhagen and his colleagues at Karolinska institute in Stockholm in the 1960s [11] was used.

Fine-gauge number 23 single-use disposable needles were used in combination with regular 10 cc single-use disposable plastic Becton Dickenson. Two to three dry clean slides were used for preparing the smears. All slides labeled with a glass pencil and air-dried. As routine, all smears are fixed with 95% alcohol and stained with eosin and heamotoxylin stain.

### Inclusion

### All those that had cytological reported negative for malignancy and consented were eligible for the study

Other study variables included use of contraceptives (ever used), Body Mass Index (BMI), Parity, chronic drug use, menarche, menopausal status, family history of breast cancer. These data were collected prospectively using a pretest questionnaire in a face to face interview. Parity low (1–2) and high (≥ 3).

Chronic drug use was any medicines in regular use for chronic conditions and included anti hypertensive, HAART, anti-tuberculosis medicines and bronchodilators.

Ethical approval was obtained from the College of Health Sciences, School of Medicine Research & Ethics Committee at Makerere. All participants provided informed written consent.

### Reliability and validity strategies

The histo-pathological examinations were done by three pathologists with experience in breast diseases in case of discrepancy in reporting. In cases of disagreement the final verdict was by consensus. The cytomorphologic features of proliferative breast lesions in conjunction with cytologic scoring system proposed by Masood et al. and histopathology were used as the diagnostic criteria [12-14].

Blinding: The pathologists were initially blinded from findings of the others.

Population Validity: the accessible population was comparable to the target population.

## Results

We screened 208 women attending the breast clinic with clinical features of benign breast lumps and 195 of them were enrolled for the study. The average age was 28.4 years, range (13 to 65), average height 1.59 meter (range: 1.4 to 1.7), average weight 57.7 kg (range: 38 to 87) and BMI of 23.26 kg/m^2^ (range: 18 to 25). Breast lumps average diameter was 3.1cm (range: 1 .0 to 10.0). Age was associated with having BPBD (p=0.008). The other participant characteristics such as district of origin, parity, contraceptive use are shown in Table 1.

**Table 1:** Participants’ characteristics with BPBD and non BPBD, Uganda study 2012

**Variable**	**Paramters**	**N**	**BPBD N (%)**	**Non BPBD N (%)**	**P values**
Age (years)	10-19	56	6(17.1)	50(31.3)	0.008
	20-29	70	5(14.3)	65(40.6)	
	30-39	38	13(37.1)	25(15.6)	
	40+	31	11(31.5)	20(12.5)	
BMI (kg/m^2^)	<18.5	16	3(19)	13(81)	0.194
	18.5-24.9	142	6(4)	136(96)	
	25-29.9	29	7(24)	22(76)	
	30+	8	7(88)	1(12)	
District	Kampala	7	2(5.7)	5(3.1)	0.194
	Wakiso	127	25(71.4)	102(63.8)	
	Others	61	8(22.9)	53(33.1)	
Parity	Null	72	8(22.9)	64(40.0)	0.092
	Low (1–2)	63	8(22.9)	55(34.4)	
	Multi (>3)	60	19(54.2)	41(25.6)	
Contraceptive use	Yes	65	17(48.6)	48(30.0)	0.111
	No	130	18(51.4)	112(70.0)	
Menarche	Yes	186	35(100.0)	151(94.4)	-
	No	9	0(0.00)	9(5.6)	
Menopause	Yes	21	7(20.0)	13(8.1)	0.200
	No	174	28(80.0)	147(91.1)	
Family history of breast cancer	Yes	32	4(11.4)	28(17.5)	0.649
	No	163	31(88.6)	132(82.5)	
Chronic drug use	Yes	29	6(17.1)	23(14.3)	
	No	166	29(82.9)		

Broadly 35 lesions were proliferative and 160 non proliferative.

Benign proliferative breast lesions (BPBL) were found in 35/195 (18%) women, 12 patients of those with BPBL had been using hormonal contraception, while 23 did not use hormonal contraception. The prevalence of BPBL was 6.15% and 11.79% among hormonal contraceptive users and non hormonal contraceptive users respectively (prevalence ratio of 0.525) p=0.110.

11/35 (31.4%) patients with BPBL had atypia while 24 (68.6%) patients with BPBL were without atypia. The prevalence of BPBL with atypia was 5.64% (11/195) and prevalence of BPBL without atypia was 12.31% (24/195).

111/195 patients had the histological diagnosis of fibroadenoma; this was followed by fibrocystic change in 40/195 patients. Epidermoid cysts, the third commonest diagnosis, were in 11/195 patients; fat necrosis in 9/195 patients; tuberculosis of the breast were in 4 patients and 13 patients had other diagnoses as shown in Table 2. The proliferative nature of all lesions was limited to the fibroadenoma and fibrocystic categories, 14 and 21 respectively.

**Table 2:** The distribution of histological diagnosis of breast lesions, Uganda study 2012

**Histological diagnosis**	**Frequency**	**Percentage**
Fibroadenoma	111	56.92
Fibrocystic change	40	20.51
Epidermoid cyst	11	5.64
Fat necrosis	9	4.62
Lactating adenoma	7	3.59
TB adenitis	4	2.05
Others	13	6.67
Total	195	100.00

## Discussion

In a region where more than half of the women with breast cancer are pre-menopausal and cancer treatment outcomes bismal [15,16], it was important to examine and characterize the nature of benign breast lesions encountered in routine clinical practice in Uganda. We set out to establish the prevalence of Benign proliferate breast lesions with atypia and without atypia. We found that benign breast lesions are a common presentation as is the case elsewhere [5-8]. We found that one in five of all benign breast diseases were profilerative in nature. And one in three of the benign proliferative lesions had atypia. Atypia which is considered to carry two to four fold risk for developing breast cancer [17,18], was present in 1 in 20 women with benign breast lumps. All the women with atypia were premenopausal.

In this study, 18% (35/195) were proliferative lesions. This was comparable with a study by Schnitt SJ et al., 1993, which found the prevalence of BPBD in Japan was as high as 18% among women younger than 40 years [19]. A similar finding was documented in North America [20]. Whereas disparities for breast cancer epidemiology and biology between the races has been well established, when it comes to benign lesions not much as been done in sub Saharan Africa. And the question remains about the accurate risk for breast cancer carried by black women with proliferative breast lesions.

Oestrogen is known to influence proliferation of breast lesions. Oestrogen has intracellular receptors that mediate a cascade of genetic process that consequently result into protein synthesis. Estrogen has physiological effects of cell proliferation and differentiation during the cell cycle. Probably in benign proliferative breast disease the steroid hormones antagonizes cell differentiation and apotosis [21,22]. The normal proliferation of cells due to action of endogenous steroid hormones leads to the breast enlargement seen at puberty and the reproductive period. An impairment of this process leads to the development of fibrocystic change that eventually presents as breast lumps [22,23].

The proliferation of cells that lead to formation of a breast lump is usually a painless process, unless other intercurrent pathology such as milk stasis, infection of the lactiferous ducts, trauma with subsequent tissue necrosis, pressure effects of the lumps on the blood vessels and nerves occur. The atypical proliferation occurs most frequently in the post menopausal period. This is when serum oestrogen wanes, perhaps explaining the atypia [23].

The majority of the patients in this study were premenopausal women. This age category usually has high levels of serum estrogen.

Fibroadenoma was the most frequently diagnosed lesion, followed by fibrocystic change. Epidermoid cyst was the third most commonly diagnosed. Other histological diagnoses were fat necrosis, lactating adenoma and tuberculosis of the breast. Nalwanga S found that fibroadenoma was the most frequently diagnosed breast lumps followed by fibrocystic change then breast abscesses at Mulago hospital a decade ago in the year 2002 [8].

Almost all of the benign proliferative lesions were found in the fibrocystic change and fibroadenoma catergories. Though not statistically significant (due to small numbers), the non hormonal contraceptive users were twice more likely to have benign proliferative lesions as compared to users. Howard in 1979 suggested that oral contraceptives reduces the risk of benign breast diseases [24-26]. Oral contraceptive pill use was found not to be associated with BPBD before [27]. This is because the amount of estrogen and progestins in hormonal contraceptives are so low to cause BPBD. The oral contraceptive pills contain estrogen plus progesterone ranging from 20 to 50 mcg only.

There was an insignificant relationship between BMI and BPBD lesions, which concur with the finding by Freidenreich et al. [23]. Though this study clearly reveals the fact that a significant proportion of black Ugandan women have proliferative lesions that may carry significant risk for breast cancer, it wasn’t without limitations, it was hospital based and therefore generalization to the entire population may be done with caution. Errors could have occurred in the area of microscopic description, clerical aspects and coding [28]. However those minimized by following standards SoPs and involving more than one experienced pathologist. Diagnoses by FNA cytology may not be 100% accurate but have a high sensitivity of >85% and high specificity of 100% [29,30].

## Conclusions

Benign proliferative breast diseases are common, found mostly among premenopausal women. A significant proportion of BPBD had atypical proliferation. An accurate breast cancer risk estimate study for BPBD is recommended.

## Abbreviations

BPBD: Prevalence of proliferative breast disease; BPBL: Benign proliferative breast lesions; BMI: Body Mass Index; FNAC: Fine needle aspiration biopsy cytology; SoPs: Standard Operating Procedures.

## Competing interests

The authors declare that they have no competing interests.

## Authors’ contributions

OC conceived concept, collected data, participated in analysis and wrote the first draft. GM, TT, WD contributed to writing of drafts. GM performed critical reviews of manuscript. All authors read and approved the final manuscript.

## Pre-publication history

The pre-publication history for this paper can be accessed here:

http://www.biomedcentral.com/1471-2482/13/9/prepub
